# Beneficial Effects of Halogenated Anesthetics in Cardiomyocytes: The Role of Mitochondria

**DOI:** 10.3390/antiox12101819

**Published:** 2023-09-30

**Authors:** José Luis Guerrero-Orriach, María Dolores Carmona-Luque, Aida Raigón-Ponferrada

**Affiliations:** 1 Institute of Biomedical Research in Malaga, 29010 Malaga, Spain; 2 Department of Anesthesiology, Virgen de la Victoria University Hospital, 29010 Malaga, Spain; 3 Department of Pharmacology and Pediatrics, School of Medicine, University of Malaga, 29010 Malaga, Spain; 4 Maimonides Biomedical Research Institute of Cordoba (IMIBIC), University of Córdoba, 14004 Cordoba, Spain; mariadolores.carmona@imibic.org; 5 Cellular Therapy Unit, Reina Sofia University Hospital, 14004 Cordoba, Spain; 6 Cell Therapy Group, University of Cordoba, 14004 Cordoba, Spain

**Keywords:** mitochondria, sevoflurane, anesthesia, preconditioning, postconditioning, cardiac surgery, heart failure

## Abstract

In the last few years, the use of anesthetic drugs has been related to effects other than those initially related to their fundamental effect, hypnosis. Halogenated anesthetics, mainly sevoflurane, have been used as a therapeutic tool in patients undergoing cardiac surgery, thanks to the beneficial effect of the cardiac protection they generate. This effect has been described in several research studies. The mechanism by which they produce this effect has been associated with the effects generated by anesthetic preconditioning and postconditioning. The mechanisms by which these effects are induced are directly related to the modulation of oxidative stress and the cellular damage generated by the ischemia/reperfusion procedure through the overexpression of different enzymes, most of them included in the Reperfusion Injury Salvage Kinase (RISK) and the Survivor Activating Factor Enhancement (SAFE) pathways. Mitochondria is the final target of the different routes of pre- and post-anesthetic conditioning, and it is preserved from the damage generated in moments of lack of oxygen and after the recovery of the normal oxygen concentration. The final consequence of this effect has been related to better cardiac function in this type of patient, with less myocardial damage, less need for inotropic drugs to achieve normal myocardial function, and a shorter hospital stay in intensive care units. The mechanisms through which mitochondrial homeostasis is maintained and its relationship with the clinical effect are the basis of our review. From a translational perspective, we provide information regarding mitochondrial physiology and physiopathology in cardiac failure and the role of halogenated anesthetics in modulating oxidative stress and inducing myocardial conditioning.

## 1. Introduction

The association between the impact of some anesthetics on cells and target proteins within cells is a subject of intensive research. Thus, efforts are being made to understand some of the initially unintended effects of anesthetics [1]. Such is the case of the cardioprotective effects of halogenated hypnotics. Unlike intravenous hypnotics, halogenated agents exert beneficial effects in patients with heart valve and coronary diseases undergoing surgery [2,3]. The clinical effect of halogenated agents is referred to as anesthetic pre- and postconditioning. During cardiac surgery, halogenated hypnotics reduce myocardial injury resulting from the attenuation of coronary blood flow and ischemia/reperfusion. As a result, the use of inotropic drugs for heart failure during the postoperative period decreases [4].

The effects of inhalational anesthetics on the cardiomyocyte are mediated by the activation of a group of enzymatic cascades. The anesthetic activates pathway signaling via the membrane and induces a response that reduces oxidative stress. Thus, cellular homeostasis is maintained through the preservation of the mitochondrial membrane and the stabilization of ion channels within the membrane. As a result, these events prevent organelle swelling and subsequent cell apoptosis [5].

The effect of halogenated anesthetics on myocardial conditioning is mediated through membrane receptors, which trigger the overexpression of different enzymes included in pathways such as the Reperfusion Injury Salvage Kinase (RISK) and the Survivor Activating Factor Enhancement (SAFE) pathways [4,5,6,7,8,9,10,11,12], with other mediators such as protein kinase G (PKG) and Nitric Oxide (NO) to achieve their final objective of cardioprotection [13,14,15,16], based on keeping the myocardium stable thanks to the preservation of mitochondrial function [17,18]. The beneficial effect of inhaled hypnotics has been the main objective in many research studies that have demonstrated their benefit regarding the time of drug exposure [19,20,21,22,23,24,25,26].

The role of mitochondria in these processes seems fundamental, as has been exposed in several publications. An exhaustive analysis of the current evidence supporting the central role of mitochondria in anesthetic myocardial conditioning was the main objective of developing this review paper [3,4,17,18]

## 2. Mitochondria Biology

Mitochondria were first identified in 1856 by the Swiss physiologist Rudolph Albert von Kölliker [27], who observed granular structures in muscle tissue cells. Later, in 1890, Richard Altmann, a German pathologist and histologist, was the first to describe them as “elementary organisms” with an intracellular location and determined that they were structures responsible for vital cellular functions [28]. However, their name was not coined until 1898 by the German zoologist Carl Benda, who named them “mitochondria” derived from the fusion of “mitos”, thread, and “chondros”, grain, which recalls their shape [29].

**Figure 1:**
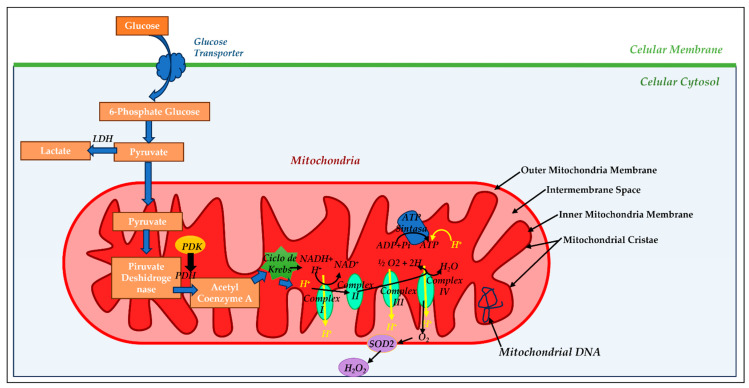
Mitochondrial morphology and oxidative phosphorylation: The outer (OMM) and inner mitochondrial membrane (IMM) are separated by an intermembrane space (IM) into which hydrogen ion (H^+^) is pumped during electron transport. The mitochondrial matrix (MM) is within the IMM and contains mitochondrial DNA (mtDNA). In the mitochondrial cristae reside the four megacomplexes of the electron transport chain (complex I, II, III, and IV) and ATP synthase enzyme, where adenosine diphosphate (ADP) and inorganic phosphate (iP) bind to produce adenosine triphosphate (ATP). Pyruvate generated in the cytosol by glycolysis (metabolic pathway by oxidation of glucose for cellular energy) enters the mitochondria and generates cellular energy or remains in the cytosol, being converted to lactate-by-lactate dehydrogenase (LDH) enzyme, if mitochondrial metabolism is inhibited. If pyruvate goes into mitochondria, it is converted to acetyl coenzyme A (CoA) by the pyruvate dehydrogenase (PDH) enzyme, the main regulator of oxidative metabolism. PDH can be inhibited by the enzyme pyruvate dehydrogenase kinase (PDK) or activated by increased mitochondrial calcium. Acetyl Co A generates energy through the Krebs cycle, and electron donors, such as Reduced Nicotin Adenine Dinucleotide (NADH) and Flavin Adenine Dinucleotide (FAD), pass into the electron transport chain to reduce molecular oxygen. This electron flow generates the chemosmotic hydrogen ion gradient that supports the ATP synthesis process. In addition, the electron flow generates reactive oxygen species (ROS), such as superoxide anion (O_2_^−^), which are converted into hydrogen peroxide molecules (H_2_O_2_) by superoxide dismutase 2 (SOD2).

Mitochondria are an intracellular organelle located in the cytoplasm of most eukaryotic cells (see Figure 1). Structurally, the mitochondria are delimited by two phospholipid membrane bilayers: the inner mitochondria membrane (IMM) and the outer mitochondria membrane (OMM), with an intermembrane space (IMS) between them housing the cytochrome C, identified as part of the electron transport chain [30]. The OMM is composed of phospholipids such as phosphatidylcholine (PC), phosphatidylinositol (PI), sphingomyelin (SM), cholesterol (chol), and proteins, and its main function is to allow the transport of material between the cell cytoplasm and mitochondria. The IMM is composed of phospholipids such as phosphatidylethanolamine (PE), phosphatidylglycerol (PG), and cardiolipin (CL), similar to the bacterial membrane and proteins [31]. The lipid distribution within the IMM and OMM is not aleatory and modulates the fluidity and membrane permeability [32]. The IMM shows a rough surface due to its high protein/lipid ratio, a ratio of 75:25, compared to the OMM and other biological membranes, and the OMM shows a smooth enriched lipid surface with very elevated fluidity [33]. The IMM phospholipid composition is related to mitochondria bioenergetic processes such as the modulation of cristae morphology, mitochondrial fusion and fission, supercomplex formation, and metabolite transport, among others [34]. The phospholipid cardiolipin (CL) is the most abundant phospholipid in the IMM, showing 15–20% of the total mitochondrial phospholipids. CL is synthesized by the mitochondria itself and plays an essential role in several fundamental mitochondrial processes, such as the formation and stability of the enzyme supercomplex (complexes I-IV) of the electron-transporting respiratory chain, dynamic control of the mitochondria, mitochondrial biogenesis, and protein import [35]. CL biosynthesis alteration has been associated with mitochondrial dysfunction in several pathological diseases, including heart failure [36]. This IMM houses an enzymatic supercomplex consisting of four protein complexes (complexes I–IV), the ATPase complex, the adenine nucleotide transporter (ANT), and many substrates such as pyruvate, a key substrate for the energetic production of mitochondria and glucose synthesis.

The IMM is where the electron exchanges of the respiratory chain take place and plays an important role in ATP synthesis processes. Finally, at the structural level, the mitochondria have an internal space called the mitochondrial matrix (MM) where a large number of enzymes participating in a wide variety of cellular metabolic processes are housed, such as the urea cycle, the Krebs cycle, or the β-oxidation of fatty acids, and favors the exchange of metabolites between the cell cytoplasm and the MM. In addition, 13 proteins that form part of the respiratory chain are located in the MM and are encoded by the mitochondrial genetic material itself, also housed in the MM, which makes the mitochondria unique compared with the rest of the cellular organelles. Mitochondrial DNA (mtDNA) is reminiscent of the prokaryotic genome from which it originates and is a circular double-stranded DNA molecule [37].

These four compartments that form the mitochondria are interdependent, and their function is perfectly coordinated to maintain cellular energy metabolism. This interdependence and coordination are observed, for example, in the functionality of a small and well-conserved protein located in the OMM, called the voltage-dependent anion-selective channel (VDAC) or mitochondrial porin, whose main function is to facilitate and regulate the flow of metabolites between the cell cytoplasm and the mitochondrial spaces [38].

At the functional level, the mitochondria are the organelle responsible for the oxidation of the products of fat, protein, and carbohydrate metabolism and their transformation into CO_2_ and H_2_O by transforming oxidative energy into chemical energy through the action of key enzymes of the respiratory chain such as NADH-dehydrogenase or NADH-ubiquinone oxidoreductase (complex I), succinate dehydrogenase (complex II), cytochrome bc1 or ubiquinone-cytochrome c oxidoreductase (complex III), and cytochrome c oxidase (complex IV). During the oxidative process, reducing molecules, such as NADH and FADH2, are generated and give up electrons to the enzymatic respiratory complexes that are transported from one oxidizing complex to the adjacent reducing complex with a higher redox potential, releasing in this process protons (H^+^) that are transferred to complex IV, as a final electron acceptor, catalyzing the oxidation of oxygen molecules to form H_2_O [39]. Throughout this process, an accumulation of H^+^ is generated in the IMS, and a release of energy, supplied indirectly to the ATP synthase protein FoF1 (complex V), pumps the H^+^ back into the MM, stabilizing the electrochemical gradient and producing 95% of the adenosine triphosphate (adenosine triphosphate: ATP) that the cell needs [40].

This chain of coupling that occurs in the mitochondria between oxidation processes and ATP formation is called oxidative phosphorylation (OxPho), a process that ensures coordination between cellular ATP needs and substrate consumption; it is fundamental for the maintenance of the physiology of various organs and tissues [41].

Another fundamental function of mitochondria is the generation of reactive oxygen species (ROS). In general terms, all oxygen-derived molecules that can live independently are identified as ROS; if they also possess one or more unpaired electrons in their orbitals, they are identified as free radicals. It has been shown that in eukaryotic cells, 80% of the O_2_ generated comes from the mitochondria at the respiratory chain level [42]. The generation of ROS by mitochondria depends exponentially on the membrane potential, i.e., when this is high, there is a reduction in the flow of electrons through the respiratory chain, which favors incomplete oxygen reductions and the consequent generation of ROS. Under physiological conditions, the detrimental effects of ROS are counteracted by antioxidant systems. Excessive production of ROS, or, in other words, a decrease in antioxidant capacity, leads to a situation of oxidative stress, which induces irreversible modifications in biological molecules and, as a consequence, the generation of irreversible tissue damage and deregulation of the processes of apoptosis and programmed cell death. The cell itself possesses antioxidant systems capable of directly eliminating ROS, such as the enzyme superoxide dismutase (SOD), glutathione peroxidase (Gpx), catalase, and thioredoxin reductase.

## 3. Mitochondrial Metabolism: Heart Function and Cardiac Failure

The healthy heart can adapt efficiently to the metabolic needs of each moment using different energy substrates such as fatty acids, lactate, ketone bodies, glucose, or amino acids, depending on the need for ATP supply. To achieve this metabolic flexibility, cardiac cells present a complex network of interrelated metabolic pathways, all capable of modifying and adapting to the physiological conditions of the moment [43].

Cardiac function is intimately linked to the metabolism of cardiac cells, and it has been shown that variations in cellular metabolism can lead to morphological and functional alterations in the heart [44,45].

The heart is an organ with a high energy demand, both for contraction and relaxation, and its function depends on the ATP supply capacity of the mitochondria included in the cardiac cells. It is for this reason that these organelles are present in high concentration in the cardiomyocytes, constituting approximately 20–40% of cell volume [46]. The ATP reserve of the heart is limited, and a reduction in ATP concentration is directly associated with oxidative stress, inflammation, and dysregulation of Ca^+2^ metabolism, leading to cardiac failure [47,48].

The main source of ATP supply in cardiac cells is the OxPho, which provides 95% of cardiomyocytes’ ATP requirements mainly through energy metabolism of substrates such as fatty acids (50%), pyruvate, ketone, and amino acids. The remaining 5% of ATP is generated through glycolysis [49,50].

To briefly summarize each of the energy substrates involved in the metabolism of the healthy heart, we can first mention fatty acids. After being absorbed by cardiac cells, fatty acids are esterified in the cell cytoplasm, forming acyl-CoA, which must be transformed into long-chain acyl-carnitine to be transported into the mitochondria [51]. Once in the mitochondria, this compound is again transformed into acyl-CoA, producing NADH and FADH2, which, through the electron transport chain, generate ATP from ADP. Fatty acids are very cost-effective substrates from an energetic point of view but less so in terms of efficiency due to the high oxygen consumption they require.

Glucose is another important energy substrate for a healthy heart. Glucose enters the cell cytoplasm through GLUT1 and GLUT4 transporters located in the plasma membrane of cardiomyocytes. Cytoplasmic glucose is transformed to pyruvate through glycolysis with the participation of lactate, this being one of the most efficient processes in ATP production in cardiomyocytes. Pyruvate enters the mitochondria through the mitochondrial pyruvate transporter and is transformed into acetyl-CoA, mainly through the action of the enzyme pyruvate dehydrogenase for ATP generation, and into oxaloacetate through the action of the enzyme pyruvate carboxylase for its contribution to the anaplerosis of Krebs cycle intermediates.

Lactate, taken up by cardiac cells through the monocarboxylic anion transporter MCT4, is converted to pyruvate by the enzyme lactate dehydrogenase, following a metabolic process similar to that of pyruvate derived from glycolysis. The role of lactate in the energy metabolism of the heart has been analyzed, and this energy substrate has been identified as its main source of pyruvate, as well as being related to signaling functions and carbon supply to the Krebs cycle [52,53,54].

Ketone bodies are another critical energy substrate for the heart [55], being easily metabolized by the heart if high ketone levels are present and are more efficient in ATP production than fatty acids. They are produced in the liver from acetyl-CoA. The main ketone body present in the heart is β-hydroxybutyrate (βOHB) in its oxidized form. The enzyme β-hydroxybutyrate dehydrogenase 1 (BDH1) is responsible for its oxidation once transported to the mitochondria, triggering a series of reactions that result in the synthesis of acetyl-CoA as input to the Krebs cycle for the final production of ATP [56].

Finally, the energetic metabolism of amino acids, a potential source of ATP production in the heart [57], generates acetyl-CoA inside the mitochondria that will be incorporated into the Krebs cycle for ATP synthesis, or succinyl-CoA for the anaplerosis of this cycle. The percentage of ATP generated by amino acid metabolism is very small, but they are very important in cardiac signaling pathways such as the insulin and mTOR pathways [58,59].

In summary, the healthy heart is an organ with high metabolic flexibility, with fatty acids being the most used substrate for ATP production, followed by lactate, ketone bodies, glucose, and, finally, amino acids.

Cardiac failure (CF) occurs as a consequence of hypertension, ischemic heart disease, valvular disease, and/or idiopathic cardiomyopathy [60]. In this pathological stage, mitochondria are unable to provide the energy production demanded by a hypertrophied heart due to several mechanisms associated with mitochondrial functionality failure, such as the following.

### 3.1. Energy Metabolism Pathways in Heart Failure

It has been widely demonstrated that the energy metabolism of the heart undergoes drastic changes when cardiac damage occurs, reducing both its metabolic flexibility [61,62] and its energy supply, with a reduction of up to 30% in the capacity for ATP synthesis when cardiac damage is in the terminal phase [63,64]. This reduction in ATP synthesis capacity is fundamentally due to the functional deterioration of the mitochondria included in the cardiac cells, associated with different processes.

### 3.2. Depletion of the ATP Reserve

There are published studies that have observed a direct association between cardiac damage and reduced myocardial ATP availability [65].

The mechanisms leading to ATP depletion are multiple; however, they all involve mitochondrial metabolism and regulation. Myocardial energy shortage not only directly compromises the relaxation and contraction of cardiac muscle fibers but also other critical ATP-dependent processes such as ion transport, including Ca^+2^ and biosynthesis of cellular components [66].

Under normal, non-pathological conditions, cellular ATP is synthesized mainly by the mitochondria through oxidative phosphorylation involving the electron transport chain located in the IMM of the mitochondria [67]. The heart contains relatively low ATP levels due to intense ATP consumption and rapid turnover [68]. Mitochondrial creatinine kinase is involved in the control of respiratory chain activity and the integration of high-energy phosphate metabolism and gives rise to phosphocreatine, which plays a key role in the maintenance of ATP content in the myocardium [69]. This enzyme can be present in its highly reactive octameric form or its less active dimeric form, with a dynamic equilibrium between the two under physiological conditions [70]. However, in heart disease, the balance between the two forms shifts towards the less active dimeric form, which impairs both respiratory control and ATP synthesis, generating a decompensation in cardiac ATP consumption. In addition, it has been shown that cardiac failure induces damage in the oxidative phosphorylation process, with a consequent reduction in cardiac ATP synthesis [71].

The adult heart obtains 50–70% of its energy through the β-oxidation of fatty acids in the mitochondria of cardiac cells [72]. Depending on nutrient availability, the myocardium can dynamically switch from the preferential use of lipids to glucose as an energy resource to maintain stable ATP production. In cardiomyocytes, this substrate selection flexibility is regulated by insulin signaling [73]. However, during heart failure, insulin signaling is downregulated or disrupted, which in turn leads to loss of metabolic flexibility.

As a result, cardiac ATP is progressively depleted, as the reduction in fatty acid oxidation is not compensated by an increase in glucose oxidation as an alternative energy source [74].

### 3.3. Oxidative Stress

Myocardial redox regulation is altered in the pathological processes derived from heart failure, leading to elevated production of both oxidative and reduced oxidative stress [75,76]. Furthermore, it has been demonstrated that there is a direct relationship between circulating levels of ROS and poor prognosis of cardiovascular damage [77] since high mitochondrial ROS production induces damage to mitochondrial DNA and defects in the electron transport chain, resulting in induction and cell death. It is important to note that the oxidative damage induced in essential mitochondrial phospholipids, such as the CL, results in both membrane surface charge density and physical property changes that impact transmembrane ion channels and ion pumps, modifying the pH and chemiosmotic gradients required for the ATP production [78]. On the other hand, Montaigne et al. evaluated the effects of myocardial contractile dysfunction derived from cardiac disease in the mitochondrial morphology and bioenergetic metabolism, and they observed a direct correlation between myocardial dysfunction and changes in mitochondrial morphology associated with impaired complex I, II, and III activity and increased oxidative stress [79].

Another important impairment at the mitochondrial level due to continuous exposure to free radicals and ROS generated in cardiac damage processes is caused by the integrity of mitochondrial DNA (mtDNA), leading to mitochondrial dysfunction [80]. These free radicals generate a large number of lesions in the mtDNA, including oxidized bases and single or double DNA strand breaks, with mutagenic or cytotoxic effects due to mismatching of bases, which generates mutations and subsequent blockages of the mtDNA replication and transcription processes, leading to great genomic instability. Repair mechanisms exist in the mitochondria themselves to repair these damages [81]; however, the frequency or accumulation of these lesions due to continued injury leads to mitochondrial dysfunction [82], deficiencies in the respiratory chain, accumulation of morphologically abnormal mitochondria [83], and an increased rate of cellular apoptosis [84]. These mtDNA damages have also been shown to affect the proper flaring of mitochondrial supercomplexes, leading to reduced ATP production and bioenergetic impairment [85].

### 3.4. Failure in Ca^2+^ Regulation Metabolism

A failure in mitochondrial Ca^2+^ metabolism is closely related to cardiac dysfunction [86]. Recent studies have shown that an insufficient Ca^2+^ supplying the mitochondria of cardiac cells causes dysfunction of enzymes involved in energy metabolism, and on the other hand, it has been observed that Ca^2+^ overload inside the mitochondria activates pathways of induction of apoptosis and cell death [87].

### 3.5. Induction of Cell Apoptosis

The loss of mitochondria functionality triggers the process of apoptosis in cardiomyocytes due to irreversible cellular damage generated in the electron transfer chain, ATP depletion, and increased oxidative stress [88].

The cardiac damage produced during the myocardial conditioning process is related to death by apoptosis of cardiac muscle cells by producing, among other processes, an increase in mitochondrial permeability and an increase in the release of cytochrome C into the cell cytoplasm, which triggers a series of irreversible events leading to apoptosis [89].

## 4. Clinical Effects of Anesthetic-Induced Myocardial Conditioning: The Role of the Mitochondria

The role of anesthetics and their interaction with cell structures have been a subject of research during the last century. A multiplicity of studies have been conducted to assess the potential beneficial effects of intravenous and halogenated hypnotics on the cardiomyocyte [2,3,4,5,6,90]. Several research studies use sevoflurane as a halogenated drug, although some studies have also been published seeking a similar effect with isoflurane and desflurane with similar results [91,92].

Ischemic pre- and postconditioning is crucial for understanding the impact of halogenated agents on the heart. These two phenomena were observed in animals exposed to short, intermittent myocardial ischemic periods. In these studies, repetitive short coronary occlusion was performed to activate cardioprotective mechanisms, which attenuated myocardial injury in subsequent longer ischemic periods. Additionally, subsequent progressive reperfusion of ischemic areas reduced cardiac ischemia/reperfusion injury. Waltier was one of the first authors to demonstrate that this technique was effective in reducing myocardial injury [6]. However, inducing episodes of ischemia entails some risks for the patient. Thus, when the duration of ischemia is extended, its therapeutic effects disappear, thereby causing cell injury and death in these areas (myocardial infarction). When the beneficial pre- and postconditioning effects of limited ischemia were confirmed, researchers investigated whether these effects could be induced pharmacologically. It is the so-called pharmacological pre/postconditioning by which halogenated agents exert similar effects as brief ischemia/reperfusion but without its associated risks [93].

In anesthesiology, these cardioprotective mechanisms have been found to be induced by halogenated agents, which partially share the mechanism of action of ischemic pre/postconditioning [5].

During cardiac surgery procedures, researchers have analyzed whether exposure to halogenated drugs confers any beneficial effects compared to intravenous drugs. The mechanism by which halogenated anesthetics induce the cardioprotective effect is related to a pool of enzymatic signaling that maintains the mitochondrial respiratory chain function, essential to energy production, through the preservation of the ATP generation as well as the reduction of oxidative damage. On the other hand, the use of propofol has been related to many disorders in the mitochondrial respiratory chain caused by the entry of different types of long-chain fatty acids. This effect has been observed in some studies carried out in animals, and they observed ventricular dysfunction due to alteration of the mitochondrial respiratory chain [94]. Additionally, propofol is a potential mitochondrial toxin that interferes with multiple mitochondrial signal pathways, including the respiratory chain. Propofol infusion syndrome, for example, mimics mitochondrial myopathies, and it is caused by the altered entry of long-chain fatty acids, impaired fatty acid oxidation, and failure of the respiratory chain in complex II [95,96]. Sevoflurane achieves the effect of reducing demands on the mitochondrial respiratory chain, which could be one of the fundamental keys to myocardial conditioning and, therefore, the volatile anesthetic-induced cardiac protection. However, molecular mechanisms, clinical aspects, the interactions with non-volatile agents, and the deprivation of the supply of oxygen and nutrients to cardiomyocytes at the beginning of acute myocardial ischemia produce damage in this type of cell that has a negative effect on their mitochondrial function and ATP production. Hanley et al. demonstrated that volatile anesthetics per se inhibit mitochondrial respiratory complex I at high concentrations [97]. Riess et al. found that sevoflurane attenuated mitochondrial respiration in a dose-dependent manner [98].

### Effect of Ischemia/Reperfusion on the Mitochondria, Oxidative Stress

Several impairments are associated with the anaerobic situation, which induces intracellular acidosis due to the accumulation of protons (H^+^), activates the transmembrane exchange of Na^+^/H^+^, produces an overload of intracellular Na^+^, and activates the Na^+^/Ca^2+^ exchange channel to correct the intracellular hypernatremia; however, it increases the calcium concentration in the mitochondria. All these procedures, together with the opening of the mitochondrial permeability transition pore (MPTP) [99,100,101,102], aggravate the detrimental effects induced by myocardial ischemia, leading to cellular apoptosis.

On the other hand, reperfusion leads to an increase in intracellular and mitochondrial Ca^2+^ produced by an instability of the cell membrane, which is added to that induced by oxidative stress in the sarcoplasmic reticulum due to alteration of the plasma membrane [103]. In myocardial reperfusion, the availability of oxygen and nutrients allows mitochondrial energization, a process which, however, by increasing mitochondrial calcium, and together with the rapid correction of pH, induces the opening of the MPTP, finally resulting in the process of cell death. Many of the factors responsible for reperfusion myocardial injury, such as mitochondrial calcium overload, oxidative stress, ATP depletion, and rapid pH correction, converge on MPTP, making it a critical factor. MPTP is a large non-selective channel that, when opened in reperfusion, allows ions and solutes up to 1,5 kDa to cross the IMM, causing mitochondrial inflammation, depolarization of the mitochondrial membrane, oxidation–phosphorylation decoupling, ATP depletion, and cell death [102,103,104,105,106]. The behavior of MPTP is completely different in the ischemia period compared to that of reperfusion; the MPTP only opens in the first minutes of reperfusion. Therefore, it is important to keep in mind that any treatment attempt to reduce the damage produced during an ischemic event and its reperfusion will have as one of the possible targets the action on this membrane pore. The opening at the time of reperfusion can also be indirectly inhibited by improving cellular bioenergetics and limiting oxidative stress, thus increasing the levels of creatine and phosphocreatine and consequently increasing the availability of ATP, which has been shown in mice overexpressing creatine [107].

In addition to MPTP-induced cell death, other mitochondria-dependent cell death pathways have been shown to contribute to cardiomyocyte death during acute myocardial ischemia/reperfusion injury.

## 5. From Troponin to Mitochondria in Anesthesia: Far Away in Clinical Setting, So Close in Cellular Mechanism

The clinical effects and mechanisms by which these effects are exerted have been extensively described in the literature. According to the literature, preconditioning induced by volatile anesthetics reduces myocardial injury, as measured by plasma Troponin I concentrations; lowers muscle/brain concentrations of creatine kinase; reduces levels of brain natriuretic peptide; attenuates free radical production; improves cardiac output, thereby requiring lower inotropic support after cardiac surgery; and shortens intensive care unit stay [2,3,4].

Cason et al. documented that exposure to isoflurane before ischemia induces anesthetic preconditioning [108]. This beneficial effect on the myocardium has been consistently observed in multiple studies. The evidence available also confirms the association between the dose and duration of the administration of these agents and their cardioprotective effects [109]. As a result of a memory effect, cardioprotective effects persist when administration is discontinued [2,90]. Intensive research has been conducted to better understand the mechanisms of action of myocardial conditioning when the anesthetic is administered during and after cardiac surgery [4]. The effector mechanisms of anesthetic pre/postconditioning share multiple pathways, all involving the protection of the mitochondria [110]. This protection is exerted through the modulation of oxidative stress, the reduction of inflammatory mediators, and the accumulation of intracellular calcium [111].

The main signaling pathways that lead to myocardial protection by maintaining mitochondrial stability are ATP-dependent potassium channels, oxygen-reactive species, apoptosis cascade, NO, and intracellular calcium overload, to name a few. These pathways mediate the protection of mitochondrial structure through their action on the mitochondrial membrane [112].

mKATP and sarcolemmal (sKATP) channels have been suggested to mediate cardioprotection through the hyperpolarization of the sarcolemma and depolarization of the internal mitochondrial membrane through the activation of intracellular signaling cascades [113,114,115]. The activation of mKATP channels attenuates the entry of mitochondrial Ca^2+^ via the Ca^2+^ uniporter. When activated, sKATP channels reduce the voltage-dependent Ca^2+^ current and limit the duration of cardiac action potential [116,117]. These events reduce Ca^2+^ entry into the sarcoplasm and mitochondria, thereby preventing injury and death induced by Ca^2+^ overload.

Numerous studies demonstrate that sevoflurane and other halogenated agents protect the myocardium from I/R injury. This protection is mediated by mitochondrial ATP-sensitive K receptors. As a result, the opening of mKATP channels favors potassium entry, reduces mitochondrial calcium overload, and favors the production of oxygen-reactive species and the activation of multiple kinases and molecular cardiac protection cascades [118,119].

Shinji Kohro et al. confirmed that volatile anesthetics activate mKATP channels [120]. This process may contribute to cardiac protection mediated by these agents. Zaugg et al. used autofluorescence live-cell imaging microscopy and a simulated model of ischemia. The authors observed that volatile anesthetics mediate cardiomyocyte protection by selectively priming mKATP channels through multiple triggering protein kinase C-coupled signaling pathways [121].

Following the administration of the halogenated agent, myocardial cell surface receptors trigger an intracellular signal mediated by other messengers (G protein) and activate adrenergic receptor-mediated signaling pathways [122]. These signaling pathways transmit the stimulus from these receptors to phospholipases C and D, thereby initiating inositol triphosphate (IP3) and diacylglycerol (DAG) synthesis. The latter releases Ca^2+^ from the reticulum and increases NO production via endothelial NO synthase [123]. This cascade of events results in the activation of protein kinase G and the different protein kinase C isoforms (PKC). PKC links cytoplasmatic and mitochondrial signaling, which influences ROS production and the opening of the mPTP and mKATP channels [124,125].

Among all signaling kinases involved in cardiovascular functions, PKC and the PKCε subgroup form a crucial pathway associated with the mKATP channel and cardiac protection. Wang et al. observed that the delayed cardioprotective effect of preconditioning increases when PKCε phosphorylation increases [126]. Similar effects were reported by Kaneda et al. and Weber et al., who confirmed the key role of the PKCε/mKATP signaling pathway in cardioprotection and the preconditioning effect of volatile anesthetics [127,128]. Preliminary results of experiments in isolated mouse heart mitochondria confirm these results, and they also demonstrated that mitochondrial matrix swelling induced by sevoflurane was not blocked by the selective PKCε inhibitor peptide εV1-2. This suggests a direct effect of sevoflurane on mKATP channels rather than via the activation of adjacent PKCε [129].

Maintaining mitochondrial matrix volume (MMV) in non-phosphorylating conditions after ischemia is important for cardioprotection induced by the opening of the mKATP channel, just as demonstrated by Riess et al. [98]. This was the first study to provide evidence that sevoflurane prevents MMV contraction during ischemia, an effect mediated by the opening of the mKATP channel. A recent study demonstrated that sevoflurane causes an increase in MMV, an effect that can be blocked by 5-HD. This finding suggests that MMV increase is mediated by the opening of the mKATP channel [113].

This event leads to downstream activation of mediators, such as tyrosine kinase, and the stimulation of mitogen-activated tyrosine kinases (MAPKs). These events induce translocation into the cell nucleus to activate specific genes that activate the following effectors:

- ATP-dependent potassium channels of the mitochondria and sarcoplasm (early preconditioning);
- Synthesis of the cytoprotective protein (late preconditioning).

The RISK and SAFE pathways are major molecular postconditioning mechanisms that converge on the mitochondrial permeability transition pore (mPTP), the final effector. Hausenloy and Yellon refer to caspase-linked anti-apoptotic pathways as RISK (Reperfusion Injury Salvage Kinase). Pharmacological activation in early reperfusion stages limits reperfusion injury. This enzymatic pathway is composed of phosphatidylinositol 3-kinases (IP3 K/Akt) and the extracellular signal-regulated kinases 1 and 2 (MEK/ERK1/2). The SAFE pathway includes the activation of the tumor necrosis factor-alpha (TNFα) and signal transducer and activator of transcription-3 (STAT-3).

The IP3 K/Akt and MEK/ERK1/2 pathway and glycogen synthase kinase 3 beta (GSK-3β) mediate the cardioprotective effect of halogenated hypnotics on the mitochondrial permeability transition pore (mPTP) [17].

Impaired mPTP function could be induced by pathological conditions, including Ca^2+^ overload and oxidative stress induced by ischemia or chronic heart failure. The opening of mPTP results in mitochondrial dysfunction, causing lipid peroxidation, ATP hydrolysis, mitochondrial matrix swelling, and, ultimately, cell death [130]. Inhibition of the opening of the mPTP pathway has been associated with preconditioning induced by volatile anesthetics. Pravdic et al. documented that isoflurane-induced preconditioning delays mPTP opening in conditions of oxidative stress in adult mouse cardiomyocytes [106]. A similar preconditioning effect induced by isoflurane was confirmed in a more recent study by Sepac et al. [131].

## 6. New Line of Research

Intensive research has been conducted on the effects of administering the volatile anesthetic isoflurane before myocardial ischemia. Thus, there is solid evidence of a reduction in myocardial injury, as measured based on troponin I concentrations, improved myocardial function, shorter ICU stay, and a decrease in morbidity and mortality, as compared to propofol [2,3,4,5].

Our research group has investigated the cardioprotective effects of halogenated agents and their effector mechanisms for 15 years. Our initial studies showed that the intraoperative administration of sevoflurane vs. propofol and their sustained administration during the postoperative period enhanced the beneficial pre/postconditioning effects induced intraoperatively by halogenated agents [3,4]. The use of inotropics and troponin blood concentrations was also found to decrease [3,4]. Our second study, a clinical trial in patients undergoing myocardial revascularization surgery, was aimed at assessing the enzymatic mechanisms by which sustained intra/postoperative administration of the halogenated agent exerted beneficial effects [88]. Patients were allocated into three groups: the first group received sevoflurane during the intra and postoperative period (SS group); the second used sevoflurane during the intraoperative period and propofol for postoperative sedation (SP group); and the third group received propofol during the intra and postoperative period (PP group). The results showed that the cardioprotective effects of sevoflurane are induced by the overexpression of the enzymes that regulate pharmacological pre/postconditioning. This effect reduced myocardial injury in the intra and postoperative sevoflurane group (lower troponin I concentrations in plasma). This event is primarily associated with the increase in the enzymes involved in the RISK and SAFE (Akt/ERK 1⁄2/STAT5) pathways, which reduce levels of cell apoptosis markers (caspases) [132]. Nitric oxide (NO) and iNOS are mediators of the activation of SAFE pathways.

NO connects the molecular and the mitochondrial mechanism of cytosol, thereby protecting the ATP-dependent K channel via the PKC-epsilon channel. This event causes iNO modulation, which reduces oxidative stress in the mitochondria. The correlation between the release of reactive oxygen species (ROS) and the effect on the reduction of mitochondrial protection induced by the halogenated agenda has already been suggested in previous studies. We also identified the action of bradykinin receptors as effectors of the limitation of oxidative injury at the cellular level and as a component involved in myocardial conditioning mechanisms via their action on the mitochondria.

We concluded that the clinical effect, which manifests in the reduction of the release of myocardial enzymes into the circulation, which was generated following cardiomyocyte apoptosis, was attenuated by the effect of anesthetics (primarily) on the mitochondria via different signaling pathways.

Transcriptional gene modulation mechanisms are essential to understanding the reduction of mitochondrial injury in pharmacological conditioning. For this reason, our latest works are focused on the initial mechanisms that control these changes and the role of small RNA, especially miRNAs, in halogenated-induced cardioprotection. A range of enzymes involved in the development of halogenated-induced cardioprotection has been associated with their expression via gene modulation, including Akt, ERK 1⁄2, and STAT proteins. These enzymes are involved in the SAFE and RISK pathways, which are effectors of mitochondrial stability. Therefore, the modulators and encoding genes of these enzymatic groups must be involved in preconditioning. miRNAs are small RNAs (19 to 25 nucleotides) that regulate protein expression through gene-specific binding and post-transcriptional mechanisms. These mechanisms either induce or inhibit the synthesis of the enzymes involved in myocardial conditioning related to mitochondrial protection and myocardial protection and remodeling. There is extensive literature on the role of miRNAs in multiple physiological or pathological cardiac processes. Gidlof et al. measured miRNAs in plasma samples of 424 patients with suspicion of acute coronary syndrome treated in a unit of cardiology. The authors found that miRNA expression levels were associated with impaired systolic function and a higher risk for mortality or cardiac failure [133]. Our group conducted a pilot study to identify the miRNAs that mediate the cardioprotective effect of halogenated anesthetics in patients at high risk for coronary ischemia. For this purpose, we investigated the potential association between the expression of a group of miRNAs and the cardioprotective effect of anesthesia-induced pre/post-conditioning. Initial encouraging results were obtained in a small group of patients, as an association was observed between the expression of some miRNAs (as measured by NGS) and the mitochondrial effect of halogenated anesthetics. The preliminary results of two different ongoing studies carried out by our research group show that preserving mitochondrial structure and function is essential to myocardial protection. The studies demonstrate that pre/post-conditioning effects are induced via different enzymatic pathways. Hence, there is a direct correlation between the overexpression of a group of miRNAs and small RNAs and anesthetic-induced pre/post-conditioning effects [90].

Our last experiment, whose methodology was recently published, was focused on cardiomyocyte cell lines. The results obtained are consistent with the clinic. Thus, the transfection of specific miRNAs involved in pre/post-conditioning modulates gene expression, thereby causing an exponential overexpression of small RNAs involved in myocardial conditioning [134].

## 7. Conclusions

The mitochondria play a major role in myocardial conditioning induced by halogenated anesthetics. Although cardiomyocyte protection is not exclusively induced by the mitochondria, the mitochondria stabilize as a result of the enzymatic cascades that modulate cell preservation pathways and variations in non-encoding Ras, which prevents cardiomyocyte apoptosis. Future advances in understanding the role of mitochondria in anesthetic-induced effects will pave the way to developing anesthetics that provide enhanced clinical benefits.
